# Reconociendo el virus del chikunguña

**DOI:** 10.7705/biomedica.5797

**Published:** 2021-06-15

**Authors:** Eliana Patricia Calvo, Edwin Darío Archila, Lady López, Jaime Eduardo Castellanos

**Affiliations:** 1 Laboratorio de Virología, Universidad El Bosque, Bogotá, D.C., Colombia Universidad El Bosque Universidad El Bosque BogotáD.C Colombia

**Keywords:** virus del chikunguña/patogenicidad, arbovirus, artritis, epidemiología, Chikungunya virus/pathogenicity, arboviruses, arthritis, epidemiology

## Abstract

El virus de chikunguña (CHIKV) es un *Alfavirus* perteneciente al grupo denominado del Viejo Mundo; estos son virus artritogénicos que causan una enfermedad febril caracterizada por artralgias y mialgias. Aunque la muerte por CHIKV es poco frecuente, la enfermedad puede llegar a ser incapacitante y generar un amplio espectro de manifestaciones atípicas, como complicaciones cardiovasculares, respiratorias, oculares, renales y dérmicas, entre otras. Cuando el dolor articular persiste por tres o más meses, da lugar a la forma crónica de la enfermedad denominada reumatismo inflamatorio crónico poschikunguña, el cual es la principal secuela de la enfermedad. Se considera que este virus no es neurotrópico, sin embargo, puede afectar el sistema nervioso central y generar secuelas graves y permanentes, principalmente, en niños y ancianos.

En África, Asia y Europa se habían reportado anteriormente brotes epidémicos por CHIKV, pero solo hasta finales del 2013 se documentó la introducción del virus a las Américas; desde entonces, el virus se ha propagado a 45 países o territorios del continente y el número de casos acumulados ascendió a cerca de dos millones en dos años.

Esta revisión describe de manera general la biología molecular del virus, sus manifestaciones clínicas, su patogénesis y las principales complicaciones posteriores a la infección. Además, reúne la información de la epidemia en Colombia y el continente americano publicada entre el 2014 y el 2020.

## Abstract (en)

The chikungunya virus (CHIKV) is an *Alphavirus* that belongs to the Old World group. These arthritogenic viruses cause a febrile illness characterized by arthralgias and myalgias.

Although fatal cases during CHIKV infection are rare, the disease may be disabling and generate a broad spectrum of atypical manifestations, such as cardiovascular, respiratory, eye, kidney, and skin complications, among others. When joint pain persists for three or more months, it results in the chronic form of the disease called post-chikungunya chronic inflammatory rheumatism, which constitutes the main disease sequel. CHIKV is not considered a neurotropic virus; however, it can affect the central nervous system, especially in children and the elderly, causing severe and permanent sequelae.

CHIKV outbreaks had been previously reported in Africa, Asia, and Europe, but the virus introduction to the American continent was documented until the end of 2013. Since then, the virus has spread to 45 countries and territories causing near two million cases in just two years. This review describes the molecular biology, clinical manifestations, pathogenesis, and significant post-infection complications of CHIKV. Additionally, it collects published information about the outbreak in Colombia and the American continent between 2014 and 2020.

La fiebre del chikunguña fue detectada por primera vez en 1952 en un hombre de la tribu Makondo en Tanzania; su nombre proviene del término utilizado por los nativos para “doblado” o “encorvado”, postura que las personas afectadas adoptan como consecuencia del fuerte dolor en las articulaciones ^1^. En los años 60, la enfermedad llegó a Asia y causó brotes en India, Sri Lanka y Tailandia; luego, se extendió a Filipinas e Indonesia en los 80 y, al final de los 90, a Malasia. Entre el 2001 y el 2009, la enfermedad resurgió en numerosos lugares del sureste asiático, en el este y sur de África, en las islas del océano Índico y por primera vez se detectó en Europa. La primera gran epidemia ocurrió en la isla La Réunion en 2005 y 2006, donde afectó a más de un tercio de la población y causó la muerte de 250 personas ^2^. A finales del 2013, se documentaron los primeros casos de la enfermedad en las Américas, en la isla Saint Martin ^3^; el número de casos acumulados desde entonces hasta finales de 2017 ascendió a cerca de 2,7 millones. Brasil, República Dominicana, Colombia, Costa Rica y Guadalupe fueron los países más afectados del continente ^4^.

La enfermedad es causada por la infección con el virus de chikunguña (CHIKV), el cual es transmitido principalmente por los mosquitos *Aedes aegypti* y *A. albopictus*. *Aedes aegypti,* también vector de los virus del dengue (DENV) y el zika (ZIKV), se encuentra distribuido en países ubicados en la zona tropical, en tanto que las zonas geográficas en las que circula *A*. *albopictus* incluye a Asia, algunos países europeos como Francia e Italia y algunas regiones de los Estados Unidos, y a más de 15 países de Centroamérica y Suramérica ^5^^,^^6^.

Aunque la infección puede ser asintomática, los síntomas típicos de la enfermedad -fiebre, mialgia y artralgia- se presentan en 70 a 95% de los casos. La viremia desaparece después de dos semanas y, con ella, la mayoría de los síntomas; sin embargo, algunos individuos desarrollan la forma crónica de la enfermedad, caracterizada por artralgias prolongadas, inclusive después de 18, 36 o 72 meses de la fase aguda ^7^^,^^8^. Hasta antes del 2006, la fiebre del chikunguña se catalogaba como “benigna”, no fatal, pero después de la gran epidemia en La Réunion, se encontró una relación directa entre la muerte y la infección, con una tasa de letalidad estimada en 1 en 1.000 infectados. Las causas comunes de muerte fueron la falla cardiaca, la falla multisistémica, la hepatitis, la encefalitis y las complicaciones neurológicas ^9^. En La Réunion también se estableció una correlación entre la gravedad de la infección y la edad, siendo los neonatos y los mayores de 65 años los más propensos a presentar los cuadros graves, así como los individuos con comorbilidades como hipertensión, diabetes, y enfermedades cardiacas y respiratorias, entre otras ^10^.

En la presente revisión, se describen de manera general el virus, su replicación, las manifestaciones clínicas asociadas con la infección y el brote epidémico colombiano ocurrido entre el 2014 y el 2015. También, se presentan resultados inéditos que demuestran que el virus no solo circuló durante el 2018 y el 2019, sino que causó una enfermedad grave en un número no despreciable de casos.

## Materiales y métodos

Se hizo una búsqueda bibliográfica con las palabras clave: chikungunya AND structure; replication; epidemiology; arthritis; mortality; chronic; Colombia; América, en las bases de datos PubMed, Scopus, y Scielo en una ventana de observación entre el 2000 y el 2020.

Como primer filtro, se revisaron el título y los resúmenes, y se incluyeron 150 artículos originales, 25 revisiones de tema, 20 reportes de casos y 10 cartas al editor. También, se consultó el Boletín Epidemiológico Semanal del Sivigila para conocer el reporte anual de casos entre el 2014 y el 2020, y tres boletines de la Organización Panamericana de la Salud, para un total de 215 registros.

Los artículos más relevantes relacionados con la estructura, el ciclo viral, la epidemiología y, especialmente, las manifestaciones clínicas, se seleccionaron para revisarlos y organizarlos sistemáticamente según cada uno de los temas tratados.

En una matriz en Microsoft Excel, se extrajeron los datos de referencia, objetivo, hallazgos principales, lugar y fecha de realización del estudio, lo que permitió organizar la información para elaborar el presente documento, el cual incluyó 113 referencias, entre las cuales 42 eran de estudios colombianos, 36 de las Américas y 38 del resto mundo.

### Virus

El CHIKV pertenece a la familia Togaviridae, género *Alfavirus,* grupo del Viejo Mundo, al que pertenecen virus artritogénicos como el virus del río Ross (RRV), el del bosque Barmah (BFV), el de Mayaro (MAYV), el O’nyongnyong (ONNV) y el de Sindbis, los cuales causan fiebre, erupción cutánea, mialgias y artralgias, principalmente ^11^.

### Genoma viral

**Figura 1:**
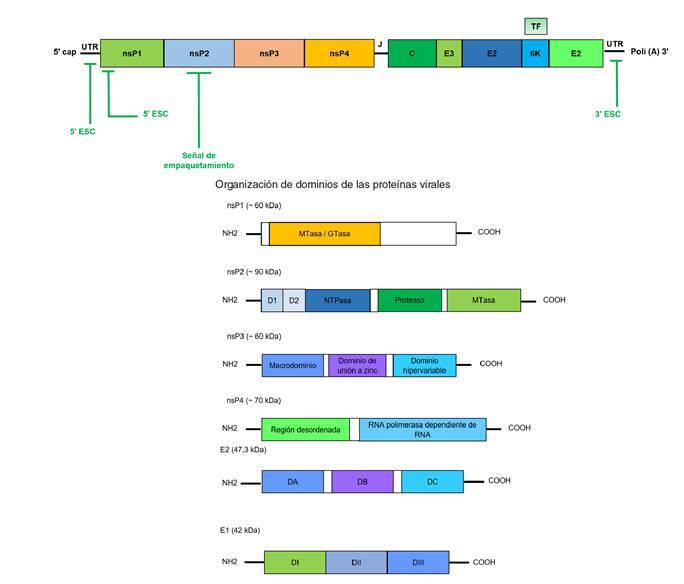
Organización del genoma. Es un ARN monocatenario de sentido positivo; presenta dos marcos abiertos de lectura separados por una región de unión no traducible (J). Se señalan las regiones no traducibles y los elementos de secuencia conservados.

Es un ARN monocatenario de sentido positivo y de 11,8 kb, aproximadamente; codifica para cuatro proteínas no estructurales, tres proteínas estructurales: la C (cápside), la E1 y la E2, y dos péptidos pequeños: E3 y 6K, dispuestos en dos marcos abiertos de lectura (*Open Reading Framework*) de 7.424 y 3.732 nucleótidos, separados entre sí por una región corta no codificante de 76 nucleótidos ^11^ (figura 1a).

Organización de dominios de las proteínas virales D1: dominio 1; D2: dominio 2; DA: dominio A; DB: dominio B; DC: dominio C; DI: dominio I; DII: dominio II; dIII: Dominio III.

El primer marco abierto de lectura codifica una poliproteína de 2.424 aminoácidos, a partir de la cual se liberan las proteínas no estructurales que dirigen la replicación del genoma. El segundo marco abierto de lectura codifica una poliproteína de 1.244 aminoácidos, a partir de la cual se liberan la proteína C, el precursor de la proteína E2 (pE2 o E2-E3), 6K y E1.

El genoma viral presenta una caperuza (*cap*) de metil-guanosina en el extremo 5´ y una cola de poliadenina en el extremo 3´. Se han descrito cuatro elementos de secuencia conservados, los cuales dan lugar a estructuras secundarias que promueven la replicación y el empaquetamiento del genoma.

Los elementos de secuencia conservados ubicados en las regiones no traducibles (*UnTranslated Region*, UTR) 5´y 3´ son complementarios, permiten el plegamiento del genoma y el acceso de la ARN polimerasa al promotor, por lo cual son esenciales para la replicación. En la región codificante de la proteína no estructural 1, se encuentra otra estructura secundaria que actúa como un potenciador de la replicación ^12^.

A partir de análisis filogenéticos realizados con el gen E1 o con el genoma completo, se identificaron tres genotipos que fueron denominados según su origen geográfico, como el del este-centro y sur de África (ECSA), al cual corresponde el primer virus aislado en Tanzania en 1952; el asiático, encontrado en Tailandia en 1960 y durante esa década en países como Vietnam, Camboya, Filipinas y el subcontinente Indio, y el de África Occidental (AO) aislado en Nigeria en 1969 y, posteriormente, en Senegal y Costa de Marfil ^2^.

Entre 1952 y el 2000, los brotes de CHIKV presentaron un patrón cíclico con periodos interepidémicos largos, de hasta 10 años, limitados a ciertas regiones de África y Asia ^2^. Entre el 2004 y el 2011, el genotipo ECSA se propagó rápidamente desde Kenia a múltiples lugares del este de África, a las islas del océano Índico, a la India, al sudeste asiático y a Europa ^13^. Estos brotes se caracterizaron por una rápida progresión y afectaron a millones de personas.

Durante el 2005 y el 2006, se documentó la gran epidemia en La Réunion ^14^, y entre el 2006 y el 2007, se notificaron en India más de un millón de casos ^15^. En 2007, se reportó la transmisión autóctona del virus en Italia ^16^ y, tres años después, en Francia ^17^.

El análisis de secuencia demostró que los virus pertenecen a un mismo linaje denominado IOL (*lndian Ocean Lineage*) y la presencia de una mutación en la proteína E1 ^13^^,^^18^. La sustitución de una alanina (A) por una valina (V) en la posición 226 (A226V), se encontró en aislamientos provenientes de La Réunion, Europa y el sudeste asiático, regiones en donde se demostró que *A. albopictus* fue el vector predominante ^18^^,^^19^. El papel de este cambio en la adaptación del virus a este mosquito se demostró en estudios *in vitro*, en los que la mutación aumentó la infección, la diseminación y la transmisión del virus por esta especie ^20^.

Solo a finales del 2013, se introdujo el CHIKV en las Américas y se propagó rápidamente a las islas del Caribe, Centroamérica y Suramérica. Entre el 2013 y el 2016, el virus se diseminó por más de 40 territorios, causando cerca de 2,7 millones de casos y ocasionando, no solo problemas de la salud en la población, sino un gran impacto en la capacidad productiva y en el desarrollo socioeconómico de las regiones más vulnerables ^21^.

El virus, perteneciente al genotipo asiático, fue detectado por primera vez en la isla Saint Martin y fue el responsable de la primera gran epidemia del continente. Aunque en el 2014 se reportó la presencia del genotipo ECSA en Brasil, su propagación explosiva no se ha documentado ^21^.

El virus encontrado en múltiples territorios presentó una gran similitud con las cepas que circularon en Indonesia, China y Filipinas en 2007, 2012 y 2013, respectivamente ^22^; no obstante, varios análisis filogenéticos evidenciaron que las secuencias americanas se separan en un grupo monofilético, el cual se denominó linaje asiático americano ^21^^,^^23^^,^^24^. Este linaje está definido por dos sustituciones: el cambio de una valina por una alanina en la posición 368 de E2 (V368A), y el de una leucina por una metionina en la posición 20 de la proteína 6K (L20M) ^21^^,^^23^. Otro rasgo característico del linaje es una duplicación de 177 nucleótidos en el extremo 3´UTR, sin impacto alguno sobre la replicación del virus en células de mamífero en los estudios *in vitro*, pero que sí le confiere una ventaja en la replicación de hasta 10 veces en células de mosquito, lo cual tendría un impacto directo sobre la transmisión del virus por el vector ^24^.

Cabe resaltar que las dos principales epidemias por CHIKV (en las islas del océano Índico y en las Américas), se han caracterizado por una explosiva propagación del virus, acompañada de cambios en el genoma, lo que le confiere una mejor replicación y un incremento en la transmisión por el vector asociado a la infección ^20^^,^^24^.

### Proteínas virales

La proteína no estructural 1 presenta una actividad de metiltransferasa y guaniltransferasa que, junto con la actividad ARN trifosfatasa de la proteína no estructural 2, se encargan de formar la caperuza (*cap*). La proteína no estructural 2 presenta, además, un dominio helicasa, actividad NTPasa y un dominio proteasa carboxi-terminal. La proteína no estructural 3 es una proteína de unión a ARN y la proteína no estructural 4 es la ARN polimerasa dependiente de ARN ^25^ (figura 1b).

La proteína de la cápside (C) tiene 261 aminoácidos y dos dominios: el C-terminal de serin-proteasa, que promueve su propia liberación de la poliproteína, y el N-terminal de interacción con ARN, que permite el empaquetamiento del genoma y la formación de la nucleocápside (NC) ^26^.

Las proteínas de la envoltura E2 y E1 son glucoproteínas transmembrana de tipo I que forman heterodímeros, los cuales, a su vez, se ensamblan en trímeros que recubren la superficie del virus en forma de espículas. La E2 facilita el reconocimiento por el receptor, la E1, la entrada a la célula vía endocitosis, y ambas permiten el proceso de salida del virus ^27^. La E3 facilita la heterodimerización de E1-E2 y evita la exposición prematura del péptido de fusión de E1 al ambiente ácido ^28^.

La proteína 6K tiene entre 55 y 60 aminoácidos y presenta dos dominios transmembranales, uno de ellos similar a los canales iónicos. La proteína TF (*TransFrame*) se origina por un corrimiento en el marco abierto de lectura durante la traducción de la proteína 6K. La proteína TF comparte el dominio N-terminal con la proteína 6K y contiene un dominio C-terminal básico conservado en otros *Alfavirus*. Estas dos proteínas intervienen en la liberación de la progenie viral, aunque su función precisa en este proceso aún se desconoce ^29^.

### Estructura del virión

El virión es esférico, con un diámetro de 60 a 70 nm, una masa molecular de 5,2 ×10^6^ Da y una densidad de 1,22 g/ml. La nucleocápside está constituida por una sola copia del ARN genómico y 240 copias de la proteína C, y está envuelta en una bicapa lipídica derivada de la célula huésped, en la que las glucoproteínas virales E1 y E2 se ordenan en un enrejado icosaédrico. Esta envoltura contiene 240 heterodímeros E1-E2 ensamblados en trímeros que dan lugar a 80 espículas ^28^.

### Ciclo de replicación

Este ciclo comienza con la unión del virus al receptor de la célula huésped mediante la glucoproteína E2. Entre los receptores descritos hasta la fecha, están la prohibitina (PHB), el TIM-1 y miembros de la familia TIM, también conocidos como receptores PVEER (*Phosphatidyl Serine Mediated Virus EntryEnhancing Receptors*), y los glucosaminoglicanos (GAG) como el heparán sulfato y la Mxra8, también conocida como DICAM, una proteína de adhesión expresada en células epiteliales, mieloides y mesenquimales ^30^^-^^33^.

Después de la unión al receptor, el virus es internalizado y liberado en endosomas; allí, el pH bajo dirige un cambio en su conformación que causa la disociación del heterodímero E1-E2, lo que lleva a la exposición del péptido de fusión en E1, el cual se inserta en la membrana endosómica formando un poro de fusión que permite la liberación de la nucleocápside en el citoplasma ^34^. La nucleocápside se une al ribosoma, lo que da inicio al desnudamiento y posterior liberación del ARN genómico (ARNg) ^28^.

Los genes no estructurales son traducidos primero, dando lugar a la poliproteína no estructural 1234; la proteína no estructural 4 es liberada rápidamente por la actividad proteasa de la proteína no estructural 2, lo que permite el ensamblaje de complejos de replicación del ARN de sentido negativo. Después de ser liberadas de la poliproteína, las proteínas maduras son responsables de la síntesis del ARNg y del ARN subgenómico (ARNsg) que sirve como plantilla para la síntesis de la poliproteína C-pE2-6K-E1.

**Figura 2:**
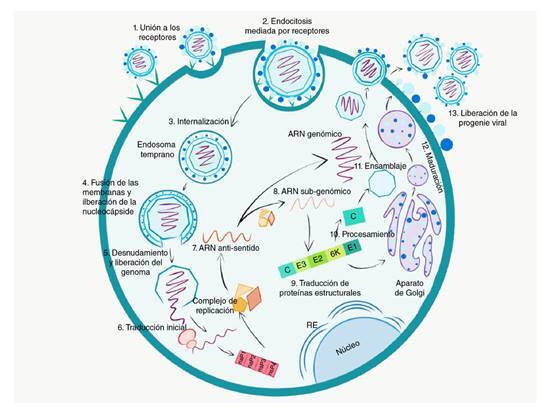
Ciclo de replicación viral: el virus ingresa a la célula por endocitosis mediada por receptores. Una vez se produce su internalización, las condiciónes ácidas del endosoma temprano producen un cambio en la conformación de la envoltura del virus que conlleva la fusión de membranas y la liberación de la nucleocápside. El genoma viral es liberado al citoplasma, las proteínas no estructurales son traducidas, se ensambla el complejo de replicación y se sintetiza un ARN antisentido, el cual es usado como plantilla para la síntesis de ARN genómico y ARN subgenómico, a partir de la cual se sintetizan las proteínas estructurales. Estas proteínas son procesadas y dirigidas a la membrana donde la partícula viral es ensamblada y liberada por gemación.

La proteína C es escindida por un proceso autocatalítico, quedando libre para encapsular el genoma y formar nuevas nucleocápsides, en tanto que la poliproteína C-pE2-6K-E1 se inserta en la membrana del retículo endoplasmático, donde son procesadas por proteasas celulares, modificadas por glucosilación, palmoilación y probablemente fosforilación y, finalmente, transportadas por la vía secretora hacia la membrana plasmática. Las pE2 y E1 se pliegan formando heterodímeros, pero antes de salir de la vía secretora, la pE2 es procesada por la furina y libera E2 y E3, clivaje necesario para producir partículas infecciosas. Las nucleocápsides ubicadas cerca de la membrana de la célula infectada interactúan con el extremo C-terminal de la proteína E2 y son envueltas por la membrana modificada por trímeros constituidos por heterodímeros E2-E1. Finalmente, la partícula viral es liberada por gemación ^35^ (figura 2).

: cápside; nsp1: proteína no estructural 1; nsp2: proteína no estructural 2¸nsp3: proteína no estructural 3¸ nsp4: proteína no estructural 4

### Tropismo celular

El virus exhibe un importante tropismo por el músculo esquelético, las articulaciones y la piel; además, es capaz de infectar una gran variedad de tipos celulares, como epitelios, endotelios, fibroblastos, osteoblastos, hepatocitos, neuronas y células de la glía ^36^^,^^37^. Ozden, *et al.*, detectaron virus en las células satélites musculares en biopsias de pacientes infectados con el CHIKV ^36^. Estas son células precursoras que persisten en el músculo esquelético maduro como células quiescentes, y se consideran responsables del crecimiento y la reparación del músculo. En el modelo de ratón, la inoculación del CHIKV en la almohadilla plantar ocasionó una pronunciada inflamación en la extremidad, acompañada de miositis, artritis, tenosinovitis y vasculitis; estos signos y el ARNg persistieron en el músculo esquelético hasta tres semanas después de la inoculación ^38^. Además, los mioblastos primarios de músculo esquelético y las líneas celulares de rabdomiosarcoma son muy permisivas con la infección ^39^^,^^40^. En las articulaciones, el virus se ha detectado en el tejido y el líquido sinovial, y los fibroblastos sinoviales, osteoblastos y condrocitos primarios son propensos a la infección ^41^^-^^44^.

Los fibroblastos podrían ser las principales células involucradas en la replicación del virus en la fase aguda, pues tanto en el modelo de ratón como en tejidos procedentes de biopsias, se detectó antígeno viral en los fibroblastos de la cápsula articular, del músculo esquelético y de la dermis. Además, las líneas celulares de fibroblastos y fibroblastos primarios son proclives a la infección ^41^^-^^44^. Aunque el CHIKV no se considera neurotrópico, la detección del virus en el cerebro y en el líquido cefalorraquídeo y la capacidad de infectar *in vitro* líneas celulares neuronales y células de la glía ^45^^-^^47^, indican que el virus puede propagarse por el sistema nervioso central y causar alteraciones que conducen a las distintas manifestaciones neurológicas ya reportadas.

En cuanto a las células del sistema inmunológico*,* Her, *et al.*^48^, sugirieron que los monocitos son las células blanco del virus durante la fase aguda y que podrían contribuir a la dispersión sistémica del virus. La detección de antígeno viral en monocitos en diversos tejidos y circulantes en un análisis histológico de tejidos *post mortem*, confirmó la propensión a la infección, lo que respalda su papel en la difusión del virus ^37^.

### Manifestaciones clínicas de la enfermedad

### Fase aguda

Entre los síntomas más comunes están la fiebre de más de 38,9 °C, artralgias, mialgias, erupción cutánea, dolor de espalda y cefalea. La aparición de la fiebre coincide con la viremia y la carga viral en sangre puede alcanzar entre 10^5^ y 10^9^ copias/ml.

Las poliartralgias, que suelen acompañarse de inflamación articular, son usualmente simétricas y afectan predominantemente las articulaciones pequeñas como los codos, las muñecas, los tobillos, las rodillas y las interfalángicas, aunque también se presentan en grandes articulaciones como los hombros y las caderas ^49^.

La erupción cutánea es transitoria y afecta principalmente las extremidades. Otras manifestaciones menos frecuentes que se han documentado son las gastrointestinales, como diarrea, vómito, náuseas y dolor abdominal, y las oculares, que pueden incluir conjuntivitis, uveítis, iridociclitis y retinitis, entre otras ^50^^,^^51^.

### Fase crónica

Cuando los dolores articulares persisten por más de tres meses, se considera alcanzada la fase crónica, conocida también como reumatismo inflamatorio crónico poschikunguña. Aunque la prevalencia puede variar entre diferentes cohortes de pacientes, un metaanálisis de la información publicada hasta junio de 2015 permitió establecer que se presenta, al menos, en el 25% de los individuos infectados ^52^. Sin embargo, en otro metaanálisis similar de la información publicada en América, se estimó que cerca del 50% de los individuos infectados podría desarrollarlo ^53^.

Esta fase sería consecuencia de la persistencia del virus en las articulaciones o de un desequilibrio entre la reacción inmunitaria proinflamatoria y la antiinflamatoria. La detección del CHIKV en macrófagos y fibroblastos de tejido sinovial en un paciente de 18 meses después de la fase aguda, permite suponer que los macrófagos podrían mantener el estado inflamatorio en las articulaciones con producción continua de IFN-α, IL-6 e IL-8, lo que causaría el deterioro del tejido ^54^. Otros mediadores inflamatorios detectados en el suero de pacientes con enfermedad crónica, son IL-1β, IL-1Ra, IL12, MCP1, GM-CSF e IL-17, citocinas implicadas en la destrucción del tejido óseo en la artritis reumatoide ^55^.

La persistencia viral también se ha demostrado en el modelo de ratón y en primates no humanos: en macacos inmunocompetentes, el virus persistió hasta 90 días después de la infección en los órganos linfoides, principalmente, y en menor grado, en músculos y articulaciones; los macrófagos fueron los principales reservorios del virus ^42^. En ratones Rag1^(-/-)^ (carentes de células T y B maduras), el genoma viral persistió en tejidos asociados a las articulaciones hasta 16 semanas después de la infección ^56^.

### Comorbilidades, formas atípicas y secuelas de la infección por el CHIKV

En el metaanálisis publicado por Badawi, *et al.*, en el 2019, se analizaron las principales comorbilidades encontradas en pacientes infectados con el CHIKV ^10^. A partir de once estudios publicados entre el 2007 y el 2017 y un total de 2.773 pacientes adultos de distintos orígenes geográficos (América y el Caribe, Europa, Asia y La Reunión), se encontró que la hipertensión es la comorbilidad más prevalente (31,3%), seguida de la diabetes (20,5%), la enfermedad cardiaca (14,8%) y el asma (7,9%). Sin embargo, solo la diabetes se encontró asociada al desarrollo de enfermedad grave (OR=1,2 y p=0,0135) (10). No obstante, después de analizar cerca de 3.000 pacientes adultos hospitalizados en Brasil, Pinto, *et al*., encontraron una asociación entre la hipertensión y las manifestaciones graves de la infección (OR=1,90; p<0,0001) ^10^.

A partir del 2005, durante la gran epidemia en La Réunion, en otras islas del océano Índico y en el subcontinente indio se comenzaron a describir manifestaciones atípicas de la enfermedad, como complicaciones cardiovasculares, respiratorias, oculares, renales y dérmicas ^1^^,^^3^^,^^9^^,^^14^. Las alteraciones neurológicas fueron señaladas como la principal causa de muerte en individuos con infección grave y se encontró una asociación entre su gravedad y la edad, siendo los recién nacidos y los mayores de 65 años los más propensos a presentar los cuadros graves ^1^^,^^9^. La tasa de letalidad por el CHIKV se calculó en 1 por 1.000 casos en La Réunion, donde se reportaron cerca de 250.000 casos y 237 muertes ^9^^,^^46^.

Durante el reciente brote en las Américas, el número de casos ascendió a cerca de 2,6 millones entre el 2013 y el 2017; Brasil, con 773.010 casos, República Dominicana, con 539.362 y Colombia, con 294.831 casos, fueron los países más afectados del continente ^4^. Las formas graves y atípicas de la enfermedad se registraron en Brasil, Colombia, Guadalupe, Martinica y México, entre otros. En varios estudios se reportaron manifestaciones atípicas, como sepsis y choque séptico, sin otro agente responsable, al igual que síntomas gastrointestinales, falla renal, dificultad respiratoria, alteraciones neurológicas y manifestaciones dérmicas diferentes a la erupción, tales como lesiones purpúricas y necrosis nasal (cuadro 1) ^37^^,^^57^^-^^72^.

**Cuadro 1:** Manifestaciones atípicas reportadas en algunos países de las Américas. Se revisaron algunos de los artículos en los que se reportaron los signos atípicos causados por la infección por el CHIKV.

País	Año	Número de pacientes evaluados	Manifestaciones atípicas	Referencias
Brasil	2016-2017	3.080 hospitalizados	Síntomas gastrointestinales: vómito, 897; náuseas, 1.081	(57)
		269 fallecidos	Petequias: 422	
			Leucopenia: 126	
Colombia	Agosto de 2014 a septiembre de 2015	295	Síntomas gastrointestinales: diarrea, 75; náuseas, 34; dolor abdominal: 23	(65)
			Manifestaciones dérmicas: úlceras en mucosa oral, 9; genital, 11	
	2015	6 pacientes pediátricos	Alteraciones neurológicas: convulsiones	(66)
			Dificultad respiratoria	
	Octubre de 2014 a agosto de 2015	109 adultos	Síntomas gastrointestinales: náuseas, 31%; vómitos, 19%	(67)
			Manifestaciones neurológicas: meningoencefalitis, 57%	
			Sangrado de mucosas, 5%	
	Septiembre de 2014 a octubre de 2015	13 casos fatales	Complicaciones renales: nefritis intersticial aguda, 11	(68)
		10 adultos		
		3 niños		
	2014	3 casos fatales	Sepsis, acrocianosis y falla orgánica múltiple	(69)
	2014	11 neonatos y lactantes	Lesiones mucocutáneas	(70)
	Septiembre de 2014 a julio de 2015	42 adultos	Alteraciones cardiovasculares: miocarditis	(71)
Guayana Francesa	Marzo de 2014 a 31 de agosto de 2015	96 pacientes	Manifestaciones neurológicas: convulsiones, 2; confusión, 4; embolia,1; encefalitis, 3; Guillain-Barré, 2; desorientación témporo-espacial, 11	(72)
		5 con enfermedad grave	Falla respiratoria: 4	
		23 con manifestaciones atípicas	Insuficiencia cardiaca: 2	
			Alteraciones hepáticas: 3	
			Pancreatitis: 2	
			Falla renal: 5	
			Sepsis: 1	
			Discapacidad muscular (rabdomiólisis): 3	
			Trombocitopenia: 1	
Guadalupe	2014	110 adultos	Veinticinco pacientes presentaron sepsis sin otro agente causante identificado.	(58)
		68 manifestaciones típicas	Manifestaciones cardiacas, respiratorias y renales	
		42 manifestaciones graves		
Martinica y Guadalupe	2013-2015	Martinica: 1.191 casos	Trastorno neurológico: 40% (convulsiones, 156; encefalitis, 38; Guillain-Barré: 13)	(59)
		Guadalupe: 630 casos	Trastornos cardiovasculares: 27%	
			Enfermedad renal 26%	
			Insuficiencia respiratoria: 22%	
			Síndrome de dolor intenso: 21%	
			Disfunción hepática: 12%	
			Manifestaciones hemorrágicas: 10%	
			Manifestaciones dérmicas: 10%	
			Trastornos digestivos: 3%	
México	Noviembre de 2014 a junio de 2015	95 pacientes	Síntomas gastrointestinales: dolor abdominal, 41; náuseas, 60; diarrea, 35	(60)
			Adenopatías: 41	
	Junio y julio de 2015	52 pacientes	Manifestaciones gastrointestinales: dolor abdominal, 20; diarrea: 15; náuseas, 34; vómito, 5	
			Petequias: 12	
			Petequias: 12	
Puerto Rico	2014	157 pacientes	Manifestaciones respiratorias: rinorrea, 58; tos, 57	(62)
		6 en unidades de cuidados intensivos	Síntomas gastrointestinales: vómito, 27; diarrea, 33; dolor abdominal,: 40	
		2 casos fatales	Petequias	
			Oculares: conjuntivitis, 90; dolor ocular, 48	
			Neurológicas: convulsiones	
	2014	27 casos fatales	Dificultad respiratoria: 8	(37)
			Cianosis: 8	
			Petequias: 12	
			Sepsis o choque séptico: 8	
			Síntomas gastrointestinales: náuseas, 7; vómito, 9; diarrea, 11	
			Dolor abdominal: 8	
Venezuela	Mayo a diciembre de 2014	4 casos graves	Choque séptico y falla multiorgánica	(64)
		3 con desenlace fatal	Lesiones purpúricas y necrosis en la región nasal	
			Acrocianosis en dedos	
	2014	3 casos con manifestaciones atípicas	Necrosis cutánea nasal y disfunción orgánica múltiple	(63)

Aunque la letalidad reportada en América es baja: 406 decesos entre el 2013 y el 2017 ^73^, en Brasil, Puerto Rico, Guadalupe, Martinica y República Dominicana, se reportó un aumento de la mortalidad durante el período de mayor afectación por el CHIKV ^74^^-^^78^. En Brasil, por ejemplo, en el 2016 se presentaron 236.287 casos y 120 muertes confirmadas; sin embargo, un análisis minucioso del total de muertes ocurridas durante ese año, comparada con los reportes del 2011 al 2013 en Pernambuco, Rio Grande del Norte y Bahía, los tres estados con las mayores tasas de incidencia del CHIKV, evidenció un aumento significativo en el número de muertes: 4.505, 1.478 y 1.517, respectivamente, esto es, 60 veces más que el reporte oficial de decesos atribuidos al virus ^75^ . En Puerto Rico, el mismo tipo de análisis estimó 1.310 muertes asociadas con la infección, en comparación con las 31 registradas en el periodo 2014-2015 ^77^. Estas cifras exponen la necesidad de análisis más precisos que den cuenta de la tasa de letalidad real del virus, dado que podría superar a la del virus del dengue, históricamente considerado como la arbovirosis de mayor impacto en América.

En cuanto a las secuelas a largo plazo de la infección, se pueden agrupar en tres categorías: reumatológicas, neurológicas y relacionadas con la salud neurosensorial ^79^. La artritis es la principal complicación reportada, pues, además del impacto en la salud del individuo, también reduce la calidad de vida y genera un significativo efecto económico negativo, dado que puede ocasionar dolor incapacitante recurrente que impide desarrollar las labores cotidianas del individuo ^80^. En el estudio de Rahim, *et al.,* de aproximadamente 1.200 individuos infectados, el 60% informó tener un grado leve de discapacidad y, el 16%, una discapacidad entre grave y moderada ^81^.

Las complicaciones neurológicas son las manifestaciones atípicas más graves y las que más frecuentemente se describen; incluyen encefalopatías y encefalitis, mielopatías y mielitis, encefalomielopatías, síndrome de Guillain- Barré, y enfermedades neurooculares, entre otras ^82^. Además, se presentan con mayor frecuencia en adultos mayores y neonatos, en los que pueden ocasionar lesiones permanentes. El análisis de la información publicada hasta el 2017 evidenció un total de 856 individuos con enfermedad neurológica asociada con el CHIKV y la encefalopatía fue la manifestación más frecuente (41% de los casos), seguido por neuropatía y alteraciones oculares, en el 9% de los casos. La encefalopatía se define como un estado mental alterado, que se refleja en confusión, desorientación, cambios en el comportamiento u otro deterioro cognitivo ^82^.

El síndrome de fatiga crónica, también conocido como encefalomielitis miálgica, es una enfermedad neurobiológica caracterizada por fatiga extrema (sin explicación aparente), acompañada por otros signos como dolor muscular o articular, cefalea, pérdida de memoria o concentración y trastornos del sueño, entre otros. La elevada prevalencia de este síndrome en pacientes seropositivos para el CHIKV comparados con individuos seronegativos, sugiere que esta podría ser una de las secuelas más importantes de la infección por el CHIKV ^83^.

El chikunguña neonatal se ha descrito en Asia, África, La Réunion y en América. Las características clínicas frecuentes en los neonatos durante la epidemia en India fueron fiebre, erupción cutánea, episodios de apnea inexplicables e hiperpigmentación difusa ^84^. En La Réunion, los neonatos desarrollaron cuadros clínicos graves de edema cerebral y, en algunos, ocasionó discapacidad persistente (deficiencia ocular y conductual o postural) ^85^. Otro estudio del mismo brote encontró que el 51% de los niños infectados al nacer tenía un retraso en el neurodesarrollo global, en comparación con los no infectados al evaluarlos a los dos años de edad. La coordinación y el lenguaje se vieron afectados con mayor frecuencia que los movimientos, la postura y la sociabilidad ^86^.

En Latinoamérica, el estudio de Torres, *et al.*^87^, incluyó 169 neonatos, 53 de El Salvador, 79 de República Dominicana y 37 de Colombia; como síntomas más representativos se reportaron fiebre (100%), dificultad para alimentarse (98,8%), irritabilidad (98,2%) y erupción cutánea (68%). En esta cohorte, se reportó un escaso número de casos con enfermedad grave caracterizada por falla respiratoria (7,7%), manifestaciones neurológicas como meningoencefalitis (7,15%) y muertes (2,3%). Por el contrario, en la primera serie de casos de transmisión vertical en Colombia, se observó una alta tasa de letalidad: 3 de 8 casos (37,5%), y manifestaciones clínicas graves, como complicaciones cardiorrespiratorias, enterocolitis necrosante, miocarditis y meningoencefalitis ^88^.

### Brote epidémico colombiano

Desde la introducción del virus en las Américas en el 2013 hasta diciembre del 2015, Colombia fue uno de los países más afectados del continente; el número de casos notificados ascendió a 275.907, es decir, el 39% de los casos notificados en el 2015 ^73^. En el 2014, se notificaron 106.763 casos; la región Caribe fue la más afectada al aportar el 61,9% de los casos y, Norte de Santander, el departamento con el mayor número de registros (24.085).

**Figura 3:**
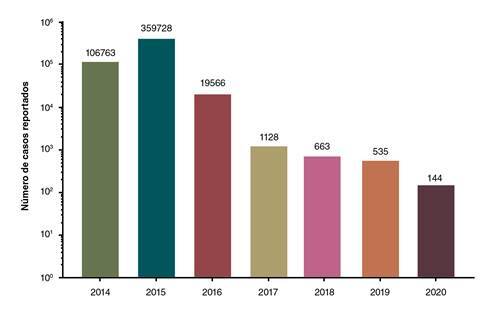
Casos notificados en Colombia en el periodo 2014-2020. Se revisaron los boletines epidemiológicos correspondientes a la semana epidemiológica 52 de cada año desde el 2014 hasta el 2019. Los datos del 2020 se tomaron del boletín epidemiológico número 33.

En el 2015, el número de notificaciones aumentó tres veces, y la región Pacífico y el Valle del Cauca fueron los más afectados, con 120.136 casos en este departamento. En ese año, en Colombia se notificó el mayor número de contagios y de muertes ^54^ asociadas con la infección del continente. A partir del 2016, el número de casos notificados al sistema de salud disminuyó significativamente, se registraron menos de 20.000 en el 2016, y en el 2018 y el 2019, se mantuvieron por debajo de 1.000 ^89^^-^^95^ (figura 3). Sin embargo, cabe destacar que, en el 2016, el virus del zika (ZIKV) ingresó al país y se diseminó en los territorios donde el DENV y el CHIKV estaban circulando, por lo que la atención y la búsqueda activa de casos se centraron en ese nuevo virus.

Debido a que los síntomas de la infección aguda por el CHIKV son similares a los causados por el DENV y a que el diagnóstico en Colombia es principalmente clínico (basado en el conjunto de signos y síntomas), es probable que el número de casos y de coinfecciones se haya subestimado. El subregistro de casos ha sido confirmado en varias regiones del país: en Girardot, departamento de Cundinamarca, por ejemplo, se notificaron 8.788 casos de CHIKV entre noviembre del 2014 y mayo del 2015; sin embargo, mediante la búsqueda activa de información en la comunidad y en cuatro instituciones de salud, se estimó un subregistro del 87%. En otras poblaciones del Caribe colombiano, como Ovejas y Corozal, departamento de Sucre, el subregistro se calculó en 48,4 y 54,2%, respectivamente ^96^.

En el 2018 y el 2019, nuestro laboratorio recolectó muestras de pacientes con síndrome febril con sospecha de infección por dengue en Cali, Villavicencio y Cartagena; a estas muestras se les hicieron las pruebas serológicas para la detección de anticuerpos IgM contra DENV, ZIKV y CHIKV, y detección del genoma viral por RT-PCR múltiple según el protocolo previamente reportado por nuestro laboratorio ^97^.

En Cali, entre junio del 2018 y julio del 2019, se evaluaron 345 pacientes pediátricos, en 270 (78,2%) de los cuales se confirmó la infección por CHIKV (243 por RT-PCR y 38 por serología). Durante todo el 2018, el departamento del Valle del Cauca reportó al sistema de salud solo 63 casos de CHIKV ^93^, en tanto que, entre junio y diciembre del mismo año, nuestro laboratorio confirmó la presencia del virus en 170 niños. De los 270 casos confirmados en el estudio, 143 correspondieron a infecciones únicas, 123 a coinfecciones con el DENV, dos a coinfecciones con el ZIKV, y hubo dos casos de infección con los tres arbovirus. En 157 (58,1%) de los casos se requirió hospitalización y siete ingresaron a cuidados intensivos. En cinco pacientes se presentaron cuadros clínicos graves de la enfermedad: tres con encefalitis, uno con miocarditis y uno con síndrome hemofagocítico, hemorragia masiva y desenlace fatal. En este último paciente se confirmó la infección única con el CHIKV mediante RT-PCR y aislamiento viral.

De los pacientes atendidos en la Clínica Meta de Villavicencio, 154 se evaluaron entre junio y diciembre del 2019: en 89 se detectó el CHIKV, en 49, el DENV, y en ocho, el ZIKV; se detectaron 35 coinfecciones, 33 casos de CHIKV-DENV, uno de CHIKV-ZIKV y otro de CHIKV-DENV-ZIKV; en 62 de los 154, se confirmó la estancia en cuidados intensivos o intermedios dada la gravedad de las manifestaciones clínicas. Durante el 2019, se notificaron al sistema nacional de vigilancia 535 casos de CHIKV, cifra que se encuentra por debajo del promedio histórico del 2017 (1.128 casos) y el 2018 (663 casos); en estos dos años, el reporte de casos para el departamento de Meta fue de 64 y 31, respectivamente, equivalentes al 5,6 y el 4,6% del total nacional (92- 94). Si consideramos un 5% de casos aportados por este departamento en el 2019, el número de casos notificados no supera los 30, casi la tercera parte de las infecciones detectadas en nuestro estudio en un periodo de seis meses.

Por último, en Cartagena, durante el segundo semestre del 2019, se evaluaron 181 pacientes pediátricos hospitalizados con diagnóstico de dengue con signos de alarma o dengue grave. Se detectó ARN viral del DENV en 89 pacientes (49%) y del CHIKV en 95 (52%); 28 de estos pacientes, aunque cumplían con el cuadro clínico de dengue con signos de alarma, fueron negativos en las pruebas serológicas y virológicas para DENV.

Aunque en el 2019 no hay reporte en el Sivigila por entidad territorial, Bolívar o Cartagena no se encuentran en la lista de territorios donde se concentró el mayor número de casos durante el año. En el 2018, Cartagena aportó solo seis casos de CHIKV al total nacional, por lo que es poco probable que en el 2019 este número haya aumentado por encima de los 95 casos detectados en el presente estudio.

También, se han documentado coinfecciones con DENV y ZIKV en otras regiones del territorio colombiano. En el estudio de Mercado, *et al.*, se detectaron 58 casos fatales asociados con el CHIKV en el periodo 2014- 2015 y se encontró una gran frecuencia de coinfecciones de DENV-CHIKV (12%) ^98^. En el análisis de Carrillo-Hernández, *et al*. ^99^, el porcentaje de coinfecciones fue del 21%. La búsqueda exhaustiva de coinfecciones durante la epidemia del ZIKV en Colombia (2015-2016) arrojó un escaso número de infecciones mixtas, solo 34 en 23.871 muestras analizadas; sin embargo, siete de estos casos tuvieron un desenlace fatal e involucraron el CHIKV ^100^.

En Colombia, la enfermedad por el CHIKV se presentó en individuos de todas las edades y estratos socioeconómicos, aunque factores como la pobreza se encontraron asociados con la infección por el CHIKV ^65^. Se registraron casos con las manifestaciones típicas de la enfermedad y resolución de la infección en pocos días, así como formas graves, especialmente en neonatos y pacientes pediátricos (66,101,10249 (49%). Las manifestaciones atípicas, como el compromiso neurológico, cardiovascular, respiratorio, renal o gastrointestinal, también se documentaron ^65^^-^^71^^,^^98^^,^^101^^-^^103^. En cuanto a la enfermedad crónica, la prevalencia reportada varió entre las diferentes cohortes, desde un mínimo de 25% en un grupo de 485 individuos y un periodo de seguimiento de 20 meses, hasta un máximo de 89% en 39 individuos y un periodo de seguimiento de 37 semanas. Además de los signos reumatológicos, se reportó rigidez matutina, edema articular y enrojecimiento articular ^104^^-^^108^.

Aunque algunos estudios han demostrado el alto costo económico de la infección por el CHIKV en nuestro país ^108^^-^^110^, el impacto real de la enfermedad aún se desconoce. Algunas variables que dificultan este análisis son el subregistro de casos, la escasez de estudios de cohorte que permitan el seguimiento de individuos infectados y la falta de estudios de seroprevalencia a nivel nacional que permitan conocer la magnitud de la epidemia. La carga de la enfermedad crónica por el CHIKV medida en años de vida ajustados o perdidos por discapacidad (AVAD) para el 2014, fue de 30,61 a 34,04 por 100.000 casos, en tanto que el consolidado de las Américas fue de 25 a 28, es decir, cerca de 1,2 veces más alto ^109^. Si tenemos en cuenta que en el 2015 el número de casos se triplicó y que los estudios de cohorte han encontrado una prevalencia media de reumatismo inflamatorio crónico poschikunguña de casi el 50% en la población infectada ^104^^-^^108^^,^^110^, es posible que estemos muy lejos de conocer la carga real de la enfermedad.

Por otro lado, en el estudio de Alvis-Zakzuk, *et al.*, que consideró el costo del tratamiento de pacientes durante la fase aguda de la enfermedad, se estimó que en los pacientes pediátricos el gasto directo de la atención (estancia hospitalaria, medicamentos y pruebas diagnósticas) fluctuó entre USD$ 121,7 en pacientes entre los 1 y los 5 años, hasta USD$ 563,8 en menores de un año ^108^. En adultos, aunque el gasto medio directo fue más bajo (USD$ 66,6), al contemplar los gastos indirectos como transporte, medicamentos y pérdida de productividad (ausencia laboral), el costo total medio estuvo cerca de los USD$ 150. Considerando solamente el número de casos reportados al sistema de salud hasta mediados del 2015, los autores estimaron un costo total medio de la infección por el CHIKV de USD$ 67 millones ^108^.

Otro aspecto importante que hasta ahora se ha pasado por alto es la caracterización virológica de los aislamientos colombianos, tanto de los recolectados durante el periodo epidémico como los de los virus circulantes en la actualidad. Aunque se han hecho varios análisis filogenéticos que indican la introducción y dispersión del genotipo asiático entre el 2014 y el 2016 (hoy reconocido como linaje asiático-americano) ^101^^,^^111^, se desconoce si en el periodo posepidémico tuvo lugar la introducción y propagación del genotipo ECSA, cuya presencia se ha descrito en Brasil y México ^2^.

La falta de conocimiento sobre el comportamiento biológico de las cepas virales colombianas y, en general, de las Américas, sumada a que los estudios sobre el ciclo de replicación y la patogénesis de la enfermedad se han hecho con aislamientos pertenecientes al linaje IOL principalmente, debería motivar la caracterización fenotípica de aislamientos colombianos del virus.

## Conclusiones

Colombia fue uno de los países del continente más afectados durante el primer brote del CHIKV en las Américas. La enfermedad afectó a individuos de todas las edades, se presentaron cuadros clínicos típicos, crónicos, graves y un número no despreciable de decesos asociados con la infección.

Aunque la descripción clínica de la enfermedad en la fase aguda fue ampliamente documentada, aún son escasos los estudios sobre la enfermedad crónica, las secuelas a largo plazo, la causa de las fatalidades, y los análisis de las consecuencias en la calidad de vida y del impacto económico generado por las dolencias después de la infección.

Esto, sumado al subregistro de casos, el escaso conocimiento sobre la circulación, la prevalencia, la introducción de nuevos genotipos en el periodo posepidémico, la ausencia de conocimiento sobre el número real de muertes asociadas con la infección y de estudios de seroprevalencia a nivel nacional para conocer la magnitud de la epidemia, lleva a pensar que el impacto real de la infección por el CHIKV en Colombia todavía se desconoce.
